# Motor cortex excitability in chronic low back pain

**DOI:** 10.1007/s00221-022-06492-7

**Published:** 2022-10-26

**Authors:** E. J. Corti, W. Marinovic, A. T. Nguyen, N. Gasson, A. M. Loftus

**Affiliations:** 1 School of Population Health, Curtin University, GPO Box U1987, Perth, WA 6845 Australia; 2 Curtin Neuroscience Research Laboratory, Curtin University, Perth, WA Australia

**Keywords:** Motor cortex, Excitability, Intracortical facilitation, Transcranial magnetic stimulation, Chronic lower back pain

## Abstract

Chronic pain is associated with dysfunctional cortical excitability. Research has identified altered intracortical motor cortex excitability in Chronic Lower Back Pain (CLBP). However, research identifying the specific intracortical changes underlying CLBP has been met with inconsistent findings. In the present case–control study, we examined intracortical excitability of the primary motor cortex using transcranial magnetic stimulation (TMS) in individuals with CLBP. Twenty participants with CLBP (*M*_*age*_ = 54.45 years, *SD*_*age*_ = 15.89 years) and 18 age- and gender-matched, pain-free controls (*M* = 53.83, *SD* = 16.72) were included in this study. TMS was applied to the hand motor area of the right hemisphere and motor evoked potentials (MEPs) were recorded from the first dorsal interosseous muscle of the contralateral hand. Resting motor threshold (rMT) and MEP amplitude were measured using single-pulse stimulation. Short interval intracortical inhibition (SICI) and intracortical facilitation (ICF) were assessed using paired-pulse stimulation. Individuals with CLBP had significantly higher rMT (decreased corticospinal excitability) and lower ICF compared to controls. No significant differences were found in MEP amplitude and SICI. These findings add to the growing body of evidence that CLBP is associated with deficits in intracortical modulation involving glutamatergic mechanisms.

## Introduction

Chronic pain is associated with altered excitability in the motor cortex (Moseley and Flor 2012; Parker et al. 2016). Motor control dysfunction is common across many chronic pain conditions, including Chronic Lower Back Pain (CLBP). There is evidence that these deficits are associated with pain-related plasticity (altered intracortical and corticospinal excitability) in both chronic and experimental pain (see reviews by Parker et al. 2016 and Sanderson et al. 2021). There is some support that individuals with CLBP and fibromyalgia demonstrate overall reductions in corticospinal excitability (Strutton et al. 2005; Mhalla et al. 2010). However, the dearth of research in this field means that the specific mechanisms underlying altered excitability in CLBP remain unclear (Schabrun and Hodges 2012).

Evidence for altered corticospinal excitability in CLBP is inconsistent (see review by Chang et al. 2018). Strutton et al. (2005) reported that people with CLBP demonstrated significantly higher motor thresholds (decreased cortical excitability) compared to controls. Conversely, Massé-Alarie et al. (2012, 2017) reported no significant difference in motor threshold and MEP amplitude between CLBP and controls. Tsao et al. (2008) also reported no significant difference in motor threshold between people with recurrent LBP and controls. The lack of consistent findings in global excitability in CLBP has led to investigating if specific intracortical mechanisms may underlie changes in excitability in CLBP.

Short interval intracortical inhibition (SICI) and facilitation (ICF) are the main mechanisms underlying cortical plasticity (Lotze and Moseley 2007). Studies in induced pain have reported changes in SICI and ICF during pain and after the removal of pain, suggesting that modulation of intracortical excitability is affected by pain perception (Brighina et al. 2011; Farina et al. 2001; Fierro et al. 2010). Farina et al. (2001) and Fierro et al. (2010) reported that changes in intracortical excitability occurred simultaneously with the onset of pain and continued as pain intensity progressed. This suggests SICI and ICF may be the key mechanisms associated with the maintenance of pain (Schabrun and Hodges 2012).

Studies examining corticospinal excitability and intracortical mechanisms in CLBP are limited. One study reported a decrease in corticospinal excitability in CLBP but did not examine intracortical mechanisms, SICI and ICF (Strutton et al. 2005). Massé-Alarie et al. (2016) reported lower SICI in people with CLBP compared to controls. There were, however, no significant differences between short ICF or cortical silent period (Massé-Alarie et al. 2016). Massé-Alarie et al. (2017) reported no difference in SICI and short ICF between people with CLBP and controls. In comparison, decreased SICI has been reported in those with chronic hand pain (Lefaucheur et al. 2006), and fibromyalgia, where both SICI and ICF levels are lower compared to controls (Mhalla et al. 2010). Although the pattern of change in these studies differs, altered motor cortex excitability is the common element of chronic pain.

Predominantly studies have focused on affected muscles (primary location of pain); however, there is some evidence to suggest that changes in excitability may be more generalised. Tagliazucchi et al. (2010) reported that chronic back pain disrupts normal activity across many cortical areas, even in brain resting state, supporting the notion that no single cortical area is responsible for the processing and evaluation of pain. This suggests that altered concentration of GABA and glutamate may reflect more of a global alteration in cortical excitability that is not purely restricted to the region representing the painful muscle (Parker et al. 2016). In addition, the First Dorsal Interosseous muscle (FDI) has previously been used in research to infer underlying cortical dynamics in diseases that do not primarily affect the motor system (Groppa et al. 2012; Rawji et al. 2020). It has been suggested that research should explore excitability in other muscles (Strutton et al. 2005).

A greater understanding of intracortical excitability in CLBP is required. It is unclear if people with CLBP have altered excitability, as indicated by responses to TMS of muscles that are not in close proximity to the site of pain (i.e., FDI). The present study sought to explore how intracortical mechanisms within the motor cortex may differ between people with CLBP and controls. The relationships between motor cortex excitability and pain-related measures were also examined.

## Methods

### Participants

Participants were recruited via convenience sampling to participate in a 5 week intervention study. The participants’ assessments at baseline form the data for this study. This study was approved by Curtin University ethics committee and all research was conducted in accordance with the Declaration of Helsinki. All participants provided written, informed consent. Inclusion in the study required a formal diagnosis of CLBP by a qualified health professional (General Practitioner or Physiotherapist) of at least 6 months (see Table 1 for demographic information and pain-related information). Individuals were screened against Transcranial Magnetic Stimulation (TMS) inclusion criteria (Rossi et al. 2011) and screened for cognitive status using the Telephone Interview for Cognitive Status—30 (TICS-30; score ≥ 18 for inclusion). Thirty-one participants met the initial inclusion criteria for participation. Eleven participants were removed from subsequent TMS analysis. Four participants did not produce reliable MEPs. Six participants had very high resting motor thresholds (rMT). One participant was excluded due to ongoing muscle activation across multiple trials. Twenty participants were included in the final data set. Control participants were recruited based on age and gender-match to the CLBP participants (*n* = 18).

**Table 1:** Demographics, pain classification, and treatment engagement

	Total		CLBP		Control	
	CLBP	Control	Males	Females	Males	Females
	(*n* = 20)	(*n* = 18)	(*n* = 11)	(*n* = 9)	(*n* = 12)	(*n* = 6)
Age	54.5 (15.9)	53.8 (16.7)	59.72 (15.9)	48.0 (14.1)	58.2 (16.5)	45.2 (14.8)
Years of education	12.5 (3.2)	14.1 (4.9)	12.1 (2.7)	13.0 (3.9)	12.0 (2.7)	13.6 (3.6)
rMT	49.8 (8.7)	42.6 (6.7)	47.8 (5.9)	52.0 (11.1)	41.2 (7.3)	38.5 (16.0)
Duration of diagnosis (years)	13.9 (13.1)	–	19.0 (15.1)	8.3 (7.9)	–	–
VAS pain average	5.0 (2.0)	–	4.6 (2.2)	5.4 (1.9)	–	–
PCS	19.7 (11.6)	–	15.8 (9.9)	24.3 (12.5)	–	–
RMDQ	12.1 (5.3)	–	13.0 (6.1)	10.9 (4.3)	–	–
CLBP classification						
Non-specific	85%	–	82%	89%	–	–
Specific	15%	–	18%	11%	–	–
Percentage taking pain medication	75%	–	55%	100%	–	–
Anti-inflammatory (celebrex, mobic, ibuprofen)*	53% ^(n = 8)^	–	67% ^(n = 4)^	44% ^(n = 4)^	–	–
Pain killer (over the counter and prescription^a^)*	67% ^(n = 10)^	–	50% ^(n = 3)^	78% ^(n = 7)^	–	–
Benzodiazepine (valium, norflex)*	27% ^(n = 4)^	–	17% ^(n = 1)^	33% ^(n = 3)^	–	–
Anti-depressants (endep)*	13% ^(n =2)^	–	17% ^(n = 1)^	11% ^(n = 1)^	–	–
Engaging in physiotherapy	50%	–	36%	67%	–	–
Past surgery	25%	–	27%	22%	–	–
Other pain management (Chiropractor)	75%	–	73%	78%	–	–
Depression and anxiety disorder	20% ^(*n* = 3)^	17% ^(*n* = 3)^	9% ^(*n* = 1)^	22% ^(*n* = 2)^	17% ^(*n* = 2)^	17% ^(*n* = 1)^
Anti-anxiety medication*	33% ^(*n* = 1)^	67% ^(*n* = 2)^	0%	50% ^(*n* = 1)^	100% ^(*n* = 2)^	0%

rMT resting motor threshold, CLBP Classification classification of chronic lower back pain based on Koes et al (2001), non-specific no radiographical injury at time of participation, Specific Radiographical evidence, Pain Average Average pain intensity 1 week prior to participation. Other Acupuncture, Chiropractor, Massage)

*Percentage based on individuals taking medication

^a^5 participants were taking prescription medication; Tramadol, Tapentadol, Lyrica and/or Codein

### Measures

Demographic and pain-related information were collected via self-report questionnaire. All CLBP participants completed TMS and clinical measures. Control participants only completed the TMS measures.

### Motor cortex excitability measures

**Fig. 1:**
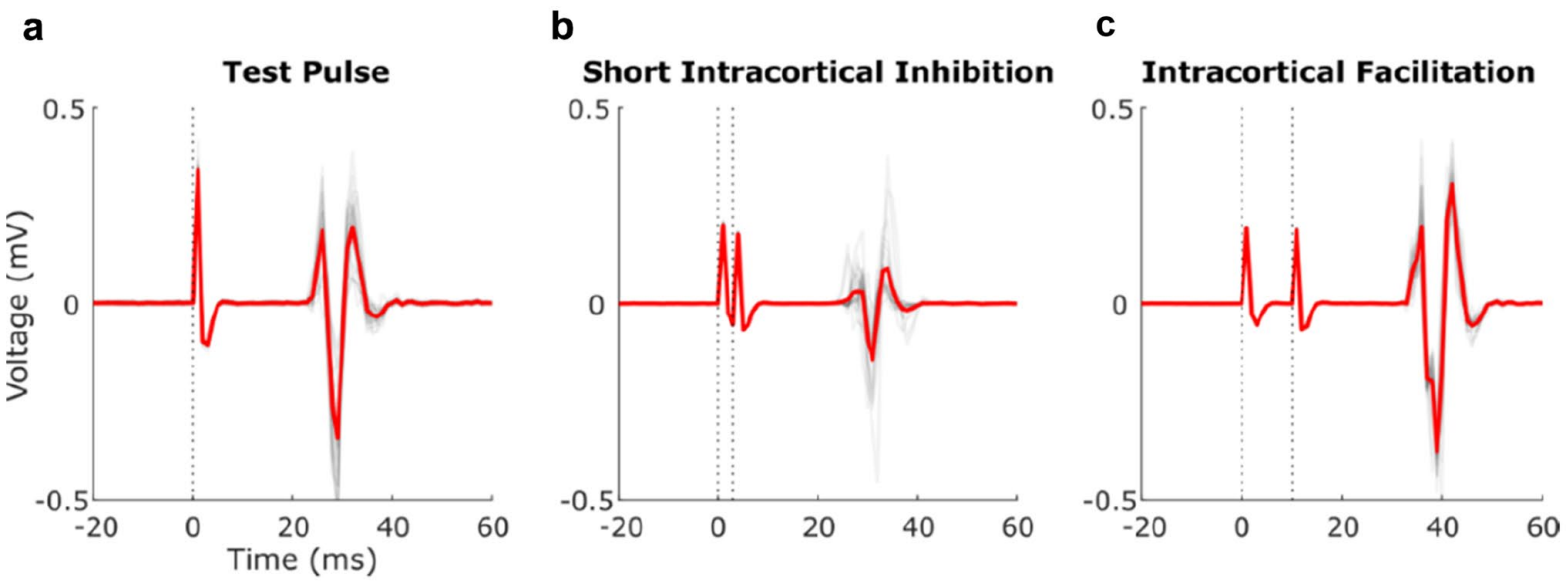
Individual average waveforms (red) of an example control participant during test pulse (**a**), ICF (**b**), and SICI configuration (**c**). Individual trials are denoted by the grey traces

EMG signals were recorded from the left FDI muscle using Ag–AgCl surface electrodes placed over the belly and tendon of the FDI (see Fig. 1). The stimulation procedures were conducted using TMS. TMS was applied using a figure-of-eight coil (90 mm in diameter) that was connected to two Magstim 200 magnetic stimulators through a Bistim module (Magstim Company Limited, UK). The motor area corresponding to the left FDI muscle was located using the 10/20 International system for electrode placement (Trans Cranial Technologies 2012). The coil was positioned over the optimal location to produce a MEP in the contralateral FDI at a 45° angle from the inter-hemispheric line, with the handle pointing toward the right-hand side, to stimulate current flow in a posterior to anterior direction.

To determine resting motor threshold (rMT), stimulation intensity started at 30% and was adjusted in 1% increments until the rMT was established. rMT was established as the lowest stimulus level that elicits MEPs greater than 50 μV in at least three of five trials, while the muscle was a rest.

SICI and ICF were measured using the paired-pulse protocol developed by Kujirai et al. (1993) SICI and ICF were assessed using a subthreshold conditioning pulse set to 80% of rMT, followed by a suprathreshold test pulse set at 120% of rMT. A moderate suprathreshold (110–120% rMT) yields the most reliable measure of SICI (Garry and Thomson 2009). The interstimulus interval was set to 3 ms for SICI and 10 ms for ICF. Fifteen trials were recorded at each interstimulus interval, and fifteen single unconditioned test pulses were also recorded (set at 120% rMT), with an 8 s interval between each trial. The order of administration was randomised. The fifteen trials for each interstimulus interval were averaged to attain a mean MEP amplitude. The mean MEP amplitude for each interstimulus interval was normalised against the participant’s mean unconditioned pulse.

### Clinical measures

#### Pain Intensity

The Short-Form McGill Pain Questionnaire (SF-MPQ) contains a 10 cm Visual Analogue Scale (VAS; scored from 0–10) and was used to assess 7 day average pain intensity in CLBP (Melzack 1987). Participants were required to mark the line at the spot they feel applied to their level of pain across the previous 7 days (Hawker et al. 2011).

#### Disability

The Roland–Morris Disability Questionnaire (RMDQ) assessed the level of disability in CLBP (Roland and Morris 1983). The RMDQ consists of 24 items assessing the impact of CLBP across multiple domains (mobility, daily activities, sleeping, mood, and appetite).

#### Pain catastrophising

The Pain Catastrophizing Scale (PCS) assessed the presence of pain catastrophising in individuals with CLBP (Sullivan et al. 1995). The PCS consists of 13 items assessing rumination, magnification, and helplessness.

### Statistical analysis

All analyses were conducted using R software (v3.5.1; R Foundation for Statistical Computing, Vienna, Austria). All trials were visually inspected and peak to peak MEP amplitudes were manually marked. Trials were excluded from analysis if visual inspection indicated noise, artifacts, or voluntary contraction, which obscured the detection of MEP amplitude, was present in the EMG signal. Trials with muscle activation were also excluded from analysis. Robust ANOVAs and independent samples T tests were conducted using WRS2 package (Mair and Wilcox 2020). Group differences in rMT, SICI, ICF, and were analysed using Yuen–Welch robust *t* test with bootstrapping, yuenbt function (nboot = 10,000). Correlations between motor cortex excitability measures (ICF and SICI) and clinical measures (pain intensity, disability, and pain catastrophising) were analysed using robust correlation, pbcor (percentage bend correlation coefficient) function.

As cortical excitability reflects the balance between excitation and inhibition, exploratory analysis was conducted to determine if the balance between SICI and ICF was related to pain. To determine the difference score between SICI and ICF, SICI MEP amplitudes were subtracted from ICF MEP amplitudes ([MEPICF/MEPcontrol]—[MEPSICI/MEPcontrol). Correlations between ICF–SICI and clinical measures (pain intensity, disability, and pain catastrophising) were analysed using robust correlation, pbcor function (see Mair and Wilcox (2020) for further details).

## Results

### Group comparison of rMT, recruitment curves, SICI and ICF

**Fig. 2:**
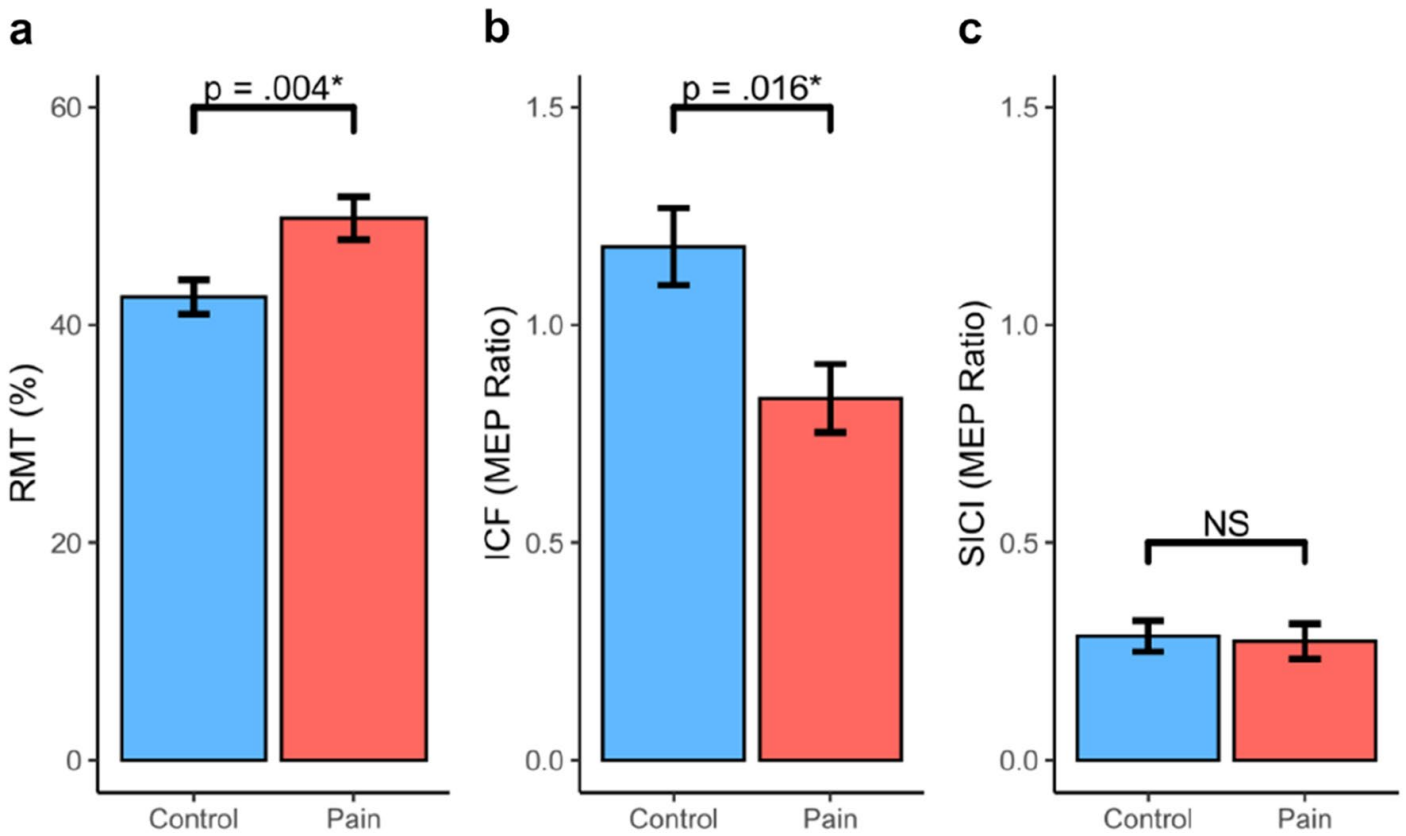
Average rMT (**a**), ICF (**b**) and SICI (**c**) MEP Ratio for CLBP and Control Group. *rMT* resting motor threshold, *ICF* intracortical facilitation, *SICI* short interval intracortical inhibition, *MEP* motor evoked potential, *MEP ratio* normalised MEP amplitude, *mV* millivolt

Analysis of rMT revealed a statistically significant difference between the CLBP and the control group (T_pb_ =  − 3.00, 95% CI [− 12.16, − 2.75], *p* = 0.004*, trimmed mean difference =  − 7.56; see Fig. 2), such that the CLBP group had a higher rMT compared to the control group. With respect to ICF and SICI, ICF was significantly lower in the pain group (T_pb_ = 2.61, 95% CI [0.09, 0.61], *p* = 0.016*, trimmed mean difference = 0.35; see Fig. 2b). However, there was no significant difference in SICI between groups (T_pb_ = 0.47, 95% CI [− 0.10, 0.16], *p* = 0.632, trimmed mean difference = 0.03; see Fig. 2c).

### Association between rMT, SICI, IFC and pain

There was no significant correlation between pain intensity and rMT (r_pb_ = 0.03, T_pb_ = 0.14, *p* = 0.891). For ICF, correlations with pain intensity (r_pb_ = 0.21, T_pb_ = 0.90, *p* = 0.379), PCS total, (r_pb_ = 0.04, T_pb_ = 0.20, *p* = 0.846) and RMDQ total (r_pb_ =  − 0.04, T_pb_ =  − 0.18, *p* = 0.856) were not statistically significant. For SICI, correlations with pain intensity (r_pb_ =  − 0.04, T_pb_ =  − 0.15, *p* = 0.884), PCS total (r_pb_ =  − 0.17, T_pb_ =  − 0.74, *p* = 0.468) and RMDQ total (r_pb_ =  − 0.005, T_pb_ =  − 0.02, *p* = 0.985) were not statistically significant.

**Fig. 3:**
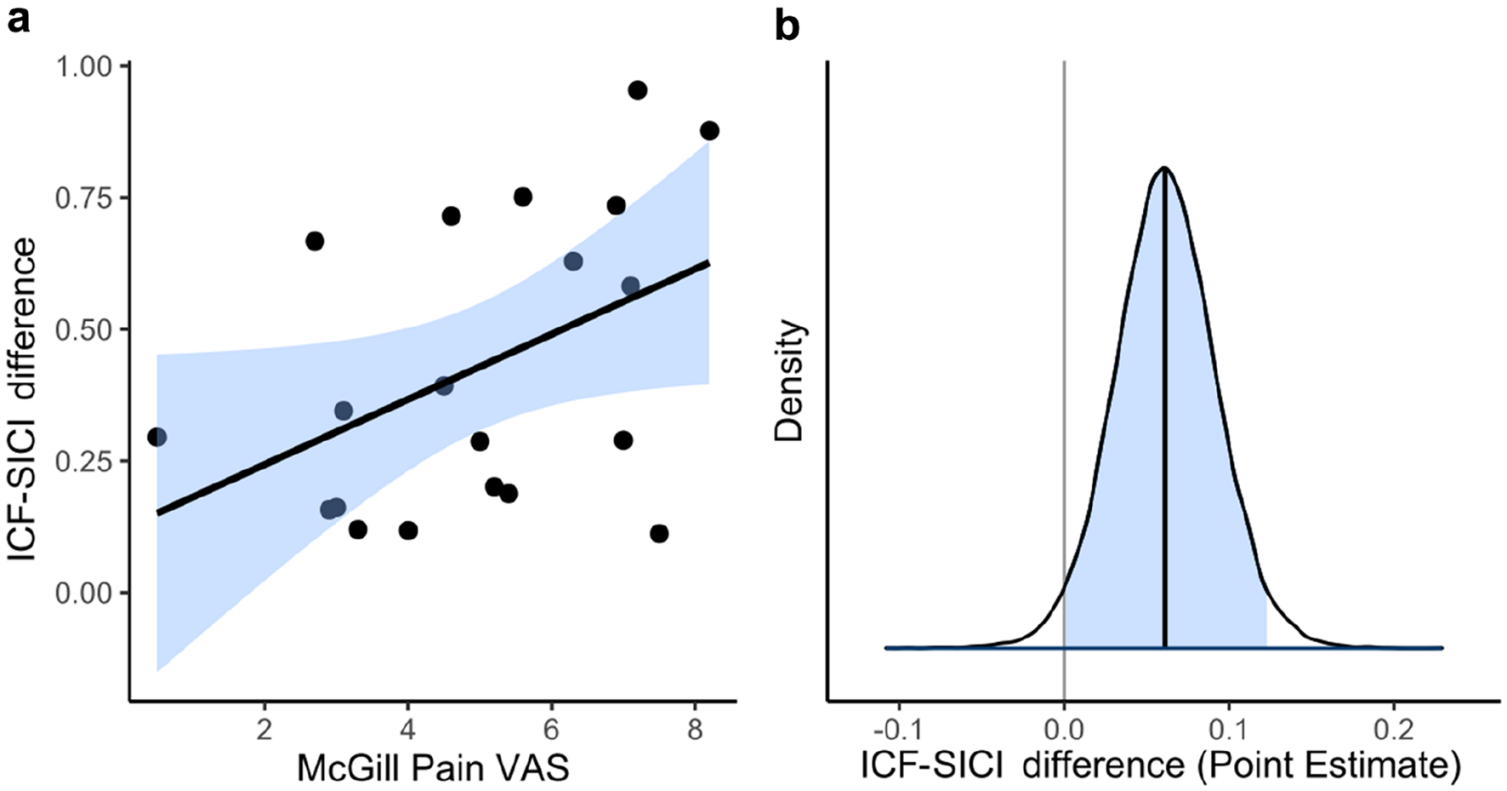
**a** Correlation between pain intensity (McGill VAS) and ICF–SICI difference (positive inhibition). **b** Bayesian posterior distribution of ICF–SICI difference for each unit increase in pain intensity (McGill VAS; 95% CI).

To explore whether pain may be explained by the balance between inhibitory and excitatory systems a correlation between pain intensity and ICF–SICI difference was conducted, and the result approached statistical significance (r_pb_ = 0.44, T_pb_ = 2.10, *p* = 0.050*; see Fig. 3a). However, correlations between ICF–SICI difference and other pain measures (PCS total, r_pb_ = 0.09, T_pb_ = 0.36, *p* = 0.720, and RMDQ total, r_pb_ = 0.02, T_pb_ = 0.07, *p* = 0.946) were non-significant.

To further examine the likelihood of the association between ICF–SICI difference and pain intensity, a Bayesian general linear model was conducted. The resulting posterior distribution (see Fig. 3b) indicated a positive association between ICF–SICI difference and pain intensity (Estimate median = 0.06 ICF–SICI difference per unit increase in pain intensity; 95% CI [0.00, 0.12]), with a positive directional probability of 97.02%, an 86% probability of significance, and a 22.27% of being large (ICF–SICI difference > 0.08).

## Discussion

The present study explored motor cortex excitability in people with CLBP, compared to pain-free, age, and gender-matched controls. The present study revealed that CLBP is associated with changes in motor cortical excitability. The CLBP group demonstrated higher rMT and lower ICF compared to controls, but there were no differences in SICI between the two groups. Individual differences in rMT, ICF and SICI were not associated with pain intensity, duration, pain sensation, use of medication, or disability in those with CLBP. Although further analysis revealed the balance between SICI and ICF (ICF–SICI difference) was associated with pain intensity.

### Resting motor threshold

Previous evidence for change in motor cortex excitability in CLBP is mixed (Chang et al. 2018). Strutton et al. (2005) reported that individuals with CLBP had a significantly higher motor threshold, which is typically indicative of decreased global excitability (Mhalla et al. 2010; Schoenen et al. 2008). Conversely, Massé-Alarie et al. (2012) reported no significant difference in motor threshold between people with CLBP and controls. The present finding of higher rMT in CLBP is consistent with Strutton et al. (2005) and with research in fibromyalgia (Mhalla et al. 2010). An increase in rMT is thought to indicate a global hypoexcitability of the corticospinal tract (Mhalla et al. 2010; Perez and Cohen 2009; Schoenen et al. 2008), which may suggest that the cortical system is less excitable in the CLBP group, compared to controls. Future research should examine recruitment curves to establish whether there are differences in MEP amplitude between people with CLBP and controls. If lower MEP amplitude in CLBP is established in the recruitment curve, this may support a global hypoexcitability of the corticospinal tract in CLBP.

### Intra-cortical facilitation and inhibition

The present study found that ICF was significantly decreased in CLBP compared to controls, a finding consistent with fibromyalgia research (Mhalla et al. 2010; Salerno et al. 2000). ICF involves excitatory glutamatergic interneurons, and lower ICF is indicative of hypoexcitability of motor circuity (Powers et al. 2014). This is the first study to show decreased ICF in people with CLBP compared to pain-free controls. Two motor cortex mapping studies reported smaller map volumes in the motor cortex in people with CLBP. Tsao et al. (2011) and Schabrun et al. (2017) reported that the smaller map volumes were indicative of lower corticomotor excitability (Massé-Alarie and Schneider 2016; Wassermann et al. 1992) and was, therefore, consistent with previous research that reported lower corticospinal excitability in CLBP (Strutton et al. 2005). Although non-significant, Massé-Alarie et al. (2017) reported an overall lower level of facilitation in people with CLBP compared to pain-free controls. Neurochemical N-Acetylaspartate, which acts on excitatory glutamate receptors, has also been shown to be lower in CLBP compared to pain-free controls (Sharma et al. 2012). Sharma et al. (2012) suggested that a reduction in N-Acetylaspartate may underlie functional motor cortex changes in CLBP. This provides further support for the hypoexcitability of the motor cortex in CLBP. In comparison with other CLBP studies, the present findings did not indicate any changes in inhibition (SICI). Strutton et al. (2005) reported decreased GABA inhibition in people with CLBP, as measured by the cortical silent period, but did not investigate any changes in SICI or ICF. Conversely, Massé-Alarie et al. (2016) reported no difference in GABA_B_ inhibition in people with CLBP but did report a decrease in GABA_A_ SICI. This discrepancy may be related to the target muscle examined (e.g., lumbar multifidus muscles vs. FDI). One possibility is that there may be distinct local and global effects on corticospinal excitability. Local effects may reflect the acute effect of pain, while global effects may indicate long-term adaptation to pain. Future research should compare the target muscle for stimulation (e.g., site of pain vs. muscles that are not in close proximity to the site of pain) to determine whether there are distinct local and global effects on corticospinal excitability in CLBP.

Hypoexcitability has been reported for other forms of chronic pain, including arthritis (Salerno et al. 2000) and fibromyalgia (Mhalla et al. 2010; Salerno et al. 2000). Although hypoexcitability may result from both spinal and supraspinal mechanisms, Mhalla et al. (2010) and Schoenen et al. (2008) suggested that hypoexcitability (as indicated by higher motor threshold) involves supraspinal mechanisms, as opposed to spinal mechanisms (Mhalla et al. 2010; Schoenen et al. 2008). The involvement of supraspinal mechanisms was supported by a lack of change in the H-reflex and dysfunctional motor control in other forms of chronic pain (Mhalla et al. 2010; Sanderson et al. 2021; Schoenen et al. 2008). Given the similar findings in the present study and frequently reported motor dysfunction in previous CLBP studies, it seems reasonable to suggest that hypoexcitability in people with CLBP may also involve supraspinal mechanisms. Establishing the involvement of supraspinal mechanisms in hypoexcitability in CLBP may be of clinical importance as there is evidence that cortical disruption contributes to, and/or maintains, chronic pain (Meier et al. 2019; Moseley and Flor 2012).

### Association between intracortical mechanisms and pain

It is known that clinical dysfunction in chronic pain and CLBP, including increased disability (Strutton et al. 2005), fatigue (Schabrun et al. 2017), depression, and catastrophising (Mhalla et al. 2010). It has been reported that the intensity of fatigue in fibromyalgia was correlated with decreased ICF, while depression and catastrophising was associated with decreased SICI (Mhalla et al. 2010). Furthermore, chronic pain studies have reported the restoration of normal SICI and ICF levels when pain was removed (Antal et al. 2010; Fregni et al. 2006a, 2006b).

The present study did not reveal significant relationships between pain intensity, disability, catastrophising, and specific measures of cortical excitability. However, the exploratory analysis revealed a relationship between the balance of facilitation and inhibition with pain intensity. Results showed that a bias toward inhibition was associated with increased pain intensity. While these results must be interpreted with caution, they do suggest that the relationship between cortical excitability and pain cannot be simply explained by a gross or singular measure (such as ICF) in isolation but may be the result of the interaction between multiple mechanisms. While the finding that increased pain is associated with SICI appears incongruent with the overall finding of decreased ICF, it is possible that lower excitability may be regulated by an increase in inhibition to keep the nervous system within a functional, dynamic range (Filmer et al. 2019). As a result, pain might be a by-product of neural dysfunction when excitation/inhibition mechanisms are not balanced.

### Limitations

There are a few limitations that must be acknowledged. The use of analgesic medications can influence cortical excitability. Benzodiazepines are reported to significantly increase SICI, as benzodiazepines increase GABA_A_ inhibitory transmission (Di Lazzaro et al. 2006). In contrast, ICF is decreased by GABA_A_ receptor agonists (such as benzodiazepines), and N-methyl-D-aspartate receptor agonists (synthetic opioids; Schwenkreis et al. 1999). While medication use was recorded in the present study, frequency of use and dosage was not documented. Although a limitation, correlations revealed that there was no relationship between medication use (use of anti-inflammatories, over-the-counter medication, prescription pain killers, benzodiazepines, or anti-depressants for pain management) and MEP amplitude. Given this, and as analgesics were not associated with changes in cortical excitability and modulation in fibromyalgia (Mhalla et al. 2010), medication use is not thought to underlie the findings of the present study. In addition, the present study did not examine motor control in CLBP. Given the involvement of motor cortical structures in movement planning and execution (Schabrun et al. 2017), it is likely that changes in motor cortex excitability may be associated with decreased motor control in people with CLBP. Future research should examine the relationship between corticospinal excitability and motor control to determine if lower facilitation is associated with lower motor control in CLBP.

## Conclusions

These findings add to the growing body of evidence that CLBP is associated with changes in intracortical excitability. While it appears changes in ICF may contribute to the pathophysiology of CLBP, future studies should examine if these changes are directly responsible for CLBP symptoms, or an indirect result of the interaction between multiple mechanisms. Nonetheless, these results may have direct clinical applications in terms of the use of neuromodulation techniques for the treatment of CLBP. Previous research has suggested that functional improvements in chronic pain are related to the restoration of altered intracortical excitability (Lefaucheur et al. 2006; Mhalla et al. 2010). Given the present findings show that altered intracortical excitability occurs in CLBP, the use of neuromodulation techniques may be of clinical significance for rehabilitation and treatment of people with CLBP.

## Data Availability

The summarised data set analysed during the current study can be made available from the corresponding author upon request.
